# Supplementary material for: Comparative multiomics analysis of cell physiological state after culture in a basket bioreactor
Source: Sci Rep. 2022 Nov 23;12:20161. doi: 10.1038/s41598-022-24687-4 (PMC9686226; doi:10.1038/s41598-022-24687-4)

23666073.1:18477415:18477526:-@NW_023666073.1:18475305:18475520:-@NW_023666073.1:18474506:1847

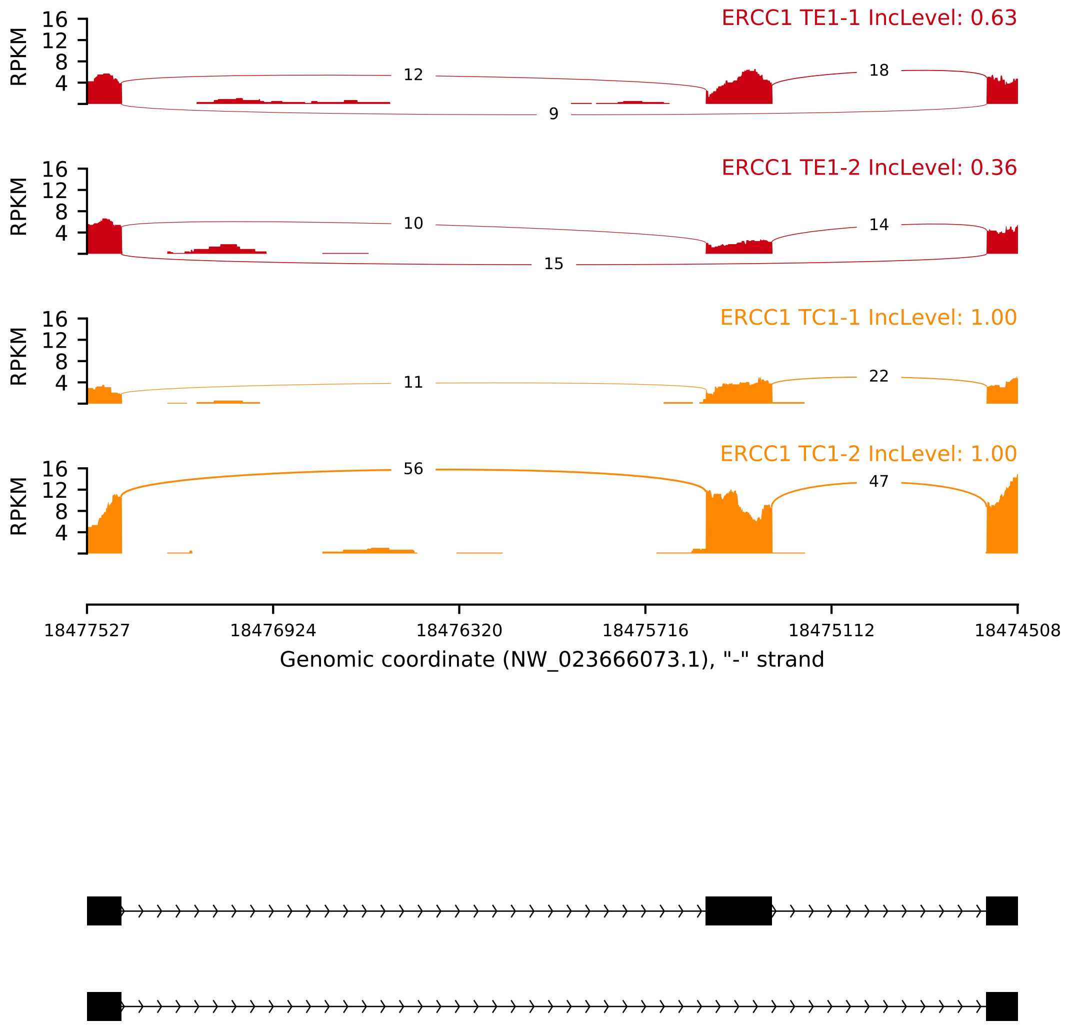

Supplement: Supplementary file 1 — Supplementary Information 1. [file 41598_2022_24687_MOESM1_ESM.zip › raw data/Transcriptomic raw data/7.AS/2.dasplot/TE1vsTC1/SE/20_ERCC1_NW_023666073.1_18477415_18477526_-@NW_023666073.1_18475305_18475520_-@NW_023666073.1_18474506_18474609_-.pdf]
